# Combined use of intravitreal bevacizumab and oral steroid treatment in three diabetic papillopathy patients: a diagnostic and treatment challenge

**DOI:** 10.3205/oc000238

**Published:** 2024-06-25

**Authors:** Burcu Taşkıran Kandeğer, Mehmet Argun, Levent Tök, Özlem Tök

**Affiliations:** 1 Konya City Hospital, Department of Ophthalmology, Konya, Turkey; 2 Suleyman Demirel University, Medicine Faculty, Department of Ophthalmology, Isparta, Turkey

**Keywords:** optic disc edema, vision loss, anti-vascular endothelial growth factor, oral steroid

## Abstract

Diabetic papillopathy (DP), a form of optic disc edema, is characterized by decreased visual acuity and mild to severe visual field defects. While there is no consensus about treatment, some publications report that intravitreal anti-vascular endothelial growth factor (anti-VEGF) injection may be beneficial. To our knowledge, however, no research reports on the effects of combining anti-VEGF injection and oral steroids in DP treatment. In this case report we present three DP cases that showed rapid improvement following therapy with intravitreal bevacizumab and oral steroids. Optic disc edemas were significantly decreased, and visual acuities were markedly increased in the first week of treatment. This report suggests that combined use of these therapies may be safely used in patients diagnosed with DP.

## Introduction

Diabetes mellitus (DM) can cause several eye complications, including a rare form of optic disc edema called diabetic papillopathy (DP). While its pathogenesis is largely unknown, DP has been reported to be significantly associated with worsening phenomena in DM patients and a small cup/disc diameter ratio [1], [2]. Hayreh et al., who described non-arteritic anterior ischemic optic neuropathy (NA-AION) in 1974, strongly argued that DP is an NA-AION subtype, not a different diagnosis [3]. Treatment remains controversial. Recent reports suggest that intravitreal anti-vascular endothelial growth factor (anti-VEGF) injections and intravitreal steroid treatments are beneficial in reducing disc swelling in DP, and Hayreh et al. reported that NA-AION patients treated with a systemic steroid showed significant improvement in visual acuity [4]. Here, we report rapid improvement following treatment with intravitreal bevacizumab and oral steroids in three patients with DP.

## Case 1

**Figure 1:**
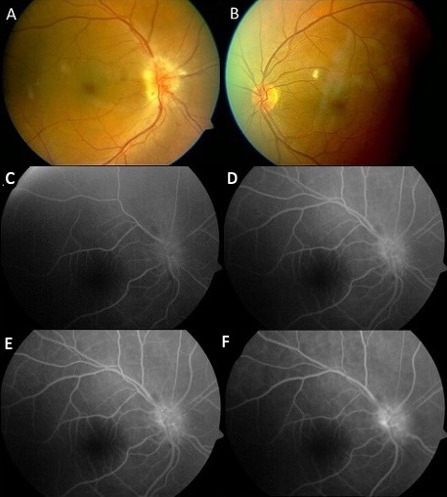
Disc edema with focal hemorrhages at the right optic disc margin (A). Cupless disc, dot and blot hemorrhages and cotton wool spot in the left eye (B). Fundus fluorescein angiography of the right eye at presentation shows a very early dye leakage from the optic disc in case 1 (C–F).

A fifty-one-year-old male patient presented with complaint of painless vision loss in the right eye. He had type 2 DM for one year. His best corrected visual acuity (BCVA) was 0.3 (Snellen chart) in the right eye and 1.0 in the left eye. The anterior segment examination was normal in both eyes except there was a relative afferent pupillary defect (RAPD) in the right eye. Color vision was preserved. The fundus examination revealed a very swollen optic disc and mild non-proliferative diabetic retinopathy in the right eye and the cup/disc ratio was under 0.1 (Figure 1A–B (Fig. 1)). Optical coherent tomography (OCT) showed a significant increase in retinal nerve fiber layer (RNFL) thickness. Fundus fluorescein angiography (FFA) showed very early-stage dye leakage from the optic disc in the right eye (Figure 1C–F (Fig. 1)). There was severe contraction of the central visual field in the right eye.

## Case 2

**Figure 2:**
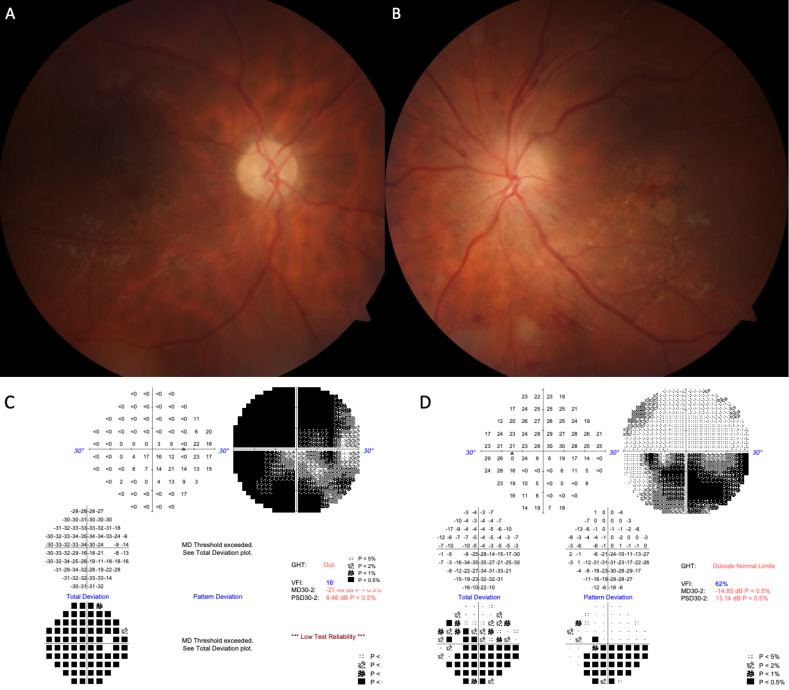
Atrophic and cupless disc, exudates accumulated in the inferior arcuate of the right eye (A). Disc edema with focal hemorrhages at the left optic disc margin (B). Severe contraction of the central visual field defect of the right eye and inferior altitudinal visual field defect of the left eye in case 2 (C–D)

A thirty-seven-year-old male patient presented with a complaint of painless vision loss in the left eye that had started four days ago. He stated that two years ago, vision loss started in his right eye too. He had type 1 DM with fifteen years history. He stated that his DM control was poor. It was learned that his HbA1c was 14.8% and 138.2 mmol/mol from the blood test results performed one week ago. His BCVA was 0.4 (Snellen chart) in the left eye and 0.3 in the right eye. There was no color vision in the left eye. The biomicroscopic examination was normal. The fundus examination revealed a very swollen optic disc and proliferative diabetic retinopathy (PDRP) in the left eye. The optic disc was atrophic in the right eye (Figure 2A–B (Fig. 2)). OCT showed a significant increase in RNFL thickness, and FFA showed early-stage dye leakage from the optic disc in the left eye. There was severe contraction of the central visual field in the right eye, and inferior altitudinal defect in the left eye (Figure 2C–D (Fig. 2)). We thought that he also had DP in his left eye previously.

## Case 3

**Figure 3:**
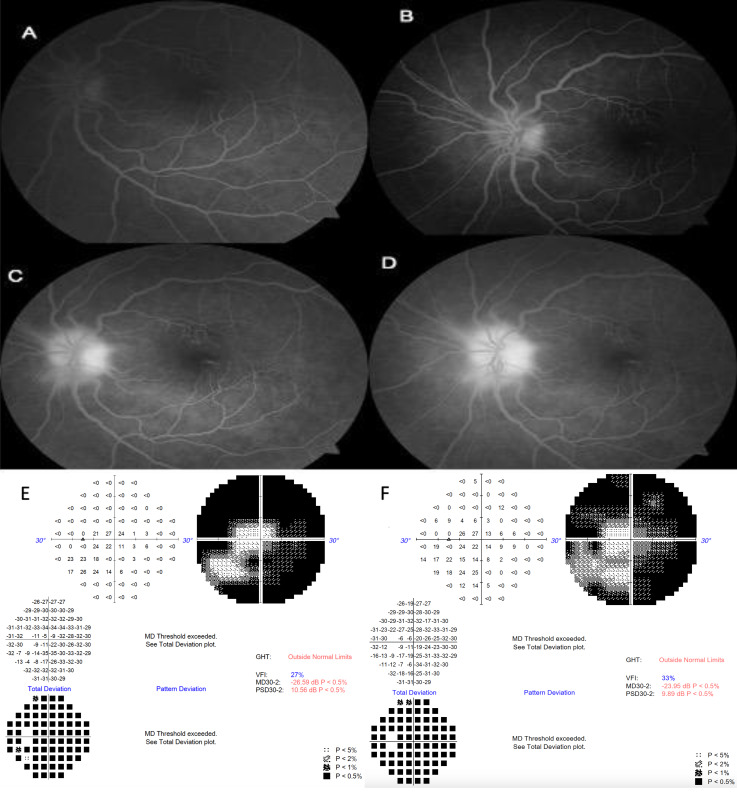
Fundus fluorescein angiography of the right eye at presentation shows a very early dye leakage from the optic disc in case 3 (A–D). Severe contraction of the central visual field defect of the left eye at the time of admission (E) and improvement in visual field after treatment (F)

A forty-eight-year-old male patient presented with complaint of painless vision loss in the right eye that had started a week earlier. He stated that nearly ten months ago, a sudden vision loss started in his right eye too. He had type 2 DM and hypertension for eleven years. His BCVA was 0.5 (Snellen chart) in the right eye and 0.4 in the left eye. The anterior segment examination was normal. The optic disc was very edematous in the right eye and pale in the left eye and the disc cup/disc ratio was 0.1. There was no color vision in the right eye. FFA showed early-stage dye leakage from the optic disc in the right eye (Figure 3A–D (Fig. 3)). There was severe contraction of the central visual field in left eye (Figure 3E (Fig. 3)).

## Further investigations and treatment

Brain and orbital magnetic resonance imaging, neurological examination, blood pressure measurements, sedimentation rate, C-reactive protein levels were normal in all patients. 

After complete examination, thorough explanation of the possible complications was given and informed consent was obtained. Intravitreal bevacizumab injection (0.5 mg/0.05 mL) (right eye for the case 1, left eye for the case 2, right eye for the case 3) and oral steroid therapy were started. 80 mg prednisone daily was started and was tapered down in 5-day steps. Patients were closely monitored for blood sugar and HbA1c levels before and after intravitreal bevacizumab and oral steroid treatment, and their insulin regimens were adjusted by an endocrinology specialist. The visual acuity increased to 0.7 (Snellen chart) in the first case, 0.9 in the second case. Visual field improvement was observed (Figure 3F (Fig. 3)), and visual acuity increased to 1.0 in the third case after a week. At 3 months follow-up, the visual acuities were stable while a slight RNFL thinning was observed in all patients. 

## Discussion

Diabetic papillopathy is considered a self-limiting disease, but visual acuity does not improve without treatment [5]. Current case reports demonstrate that intravitreal anti-VEGF injection effectively resolves optic disc edema in DP patients, suggesting that increased VEGF secretion may play a role in the condition’s pathogenesis [6], [7]. Hyperfluorescence in FA reflects the fluorescein leakage associated with the breakdown of the blood-retinal barrier. Funatsu et al. found that intravitreal VEGF levels were elevated in patients with hyperfluorescent diabetic macular edema [8]. Early optic disc hyperfluorescence in FA may explain why anti-VEGF treatment is efficacious. 

Feng et al. reported that intravitreal steroid treatment may rapidly improve the vision of DP patients. However, they also expressed concerns about the safety of this therapy [9]. Intravitreal steroids are known to significantly increase intraocular pressure, and the optic disc head, which is already hypoxic, is likely to be adversely affected by such a pressure increase. According to research by Hayreh et al., oral steroids provide faster resolution of disc edema and consequently better blood flow in the capillaries of the crowded and congested disc, preserving axonal functions in NA-AION patients [4].

Uniquely, the cases in this study were treated with a combination of intravitreal bevacizumab and oral steroids. While providing beneficial anti-edema effects, oral steroids may be safer for the eyes, as they are less likely to increase intraocular pressure. However, larger clinical studies are needed to determine the efficacy and safety of this combined treatment for DP and even NA-AION.

## Notes

### Authors’ ORCIDs

- Burcu Taşkıran Kandeğer: 0000-0002-4636-6767
- Mehmet Argun: 0000-0002-6877-4884
- Levent Tök: 0000-0003-0819-915X
- Özlem Tök: 0000-0002-1778-5233

### Patient consent

The patients described in this case report consented in written form to publication of all data and images related to the case.

### Competing interests

The authors declare that they have no competing interests.
